# Time Trend of Multiple Myeloma and Associated Secondary Primary Malignancies in Asian Patients: A Taiwan Population–Based Study

**DOI:** 10.1371/journal.pone.0068041

**Published:** 2013-07-02

**Authors:** Huey-En Tzeng, Cheng-Li Lin, Chun-Hao Tsai, Chih-Hsin Tang, Wen-Li Hwang, Ya-Wen Cheng, Fung-Chang Sung, Chi-Jung Chung

**Affiliations:** 1 Graduate Institute of Medicine, China Medical University, Taichung, Taiwan; 2 Division of Hematology/Oncology, Taichung Veterans General Hospital, Taichung, Taiwan; 3 Management Office for Health Data, China Medical University Hospital, Taichung, Taiwan; 4 Department of Public Health, China Medical University, Taichung, Taiwan; 5 Department of Orthopaedics, China Medical University Hospital, Taichung, Taiwan; 6 Department of Pharmacology, School of Medicine, China Medical University and Hospital, Taichung, Taiwan; 7 The Institute for Cancer Biology and Drug Discovery, College of Medical Science and Technology, Taipei Medical University, Taipei, Taiwan, Republic of China; 8 Department of Health Risk Management, China Medical University, Taichung, Taiwan; 9 Department of Medical Research, China Medical University Hospital, Taichung, Taiwan; Cardiff University, United Kingdom

## Abstract

Studies involving second malignancies in patients with multiple myeloma are limited for the Asian population. Using data from population-based insurance claims, we assessed the risk of developing secondary malignancies after multiple myeloma, in particular hematologic malignancies. A retrospective cohort study was conducted in 3970 patients with newly diagnosed multiple myeloma from the registry of catastrophic illnesses between 1997 and 2009. A total of 15880 subjects without multiple myeloma were randomly selected as comparisons from the insured population, frequency-matched based on gender, age, and the date of diagnosis. The incidence of secondary malignancies was ascertained through cross-referencing with the National Cancer Registry System. The Cox proportional hazards model was used for analyses. The incidence of multiple myeloma in the insured population increased annually. The overall incidence of secondary malignancy was lower in the multiple myeloma cohort than in the comparison cohort (93.6 vs. 104.5 per 10,000 person-years, IRR = 0.90, 95% CI = 0.78–1.04). The incidence of hematologic malignancies was 11-fold greater for multiple myeloma patients (47.2 vs. 4.09 per 10,000 person-years) with an adjusted HR of 13.0 (95% CI = 7.79–21.6) compared with the comparison cohort. The relative risk of secondary malignancy was also strong for myeloid leukemia (21.2 vs. 1.36 per 10,000 person-years). Gender- and age-specific analysis for secondary hematologic malignancies showed that males and patients with multiple myeloma <60 years of age had a higher risk of secondary malignancy than females and patients with multiple myeloma >60 years of age. In conclusion, patients with multiple myeloma, especially younger patients, are at a high risk of hematologic malignancies.

## Introduction

Multiple myeloma (MM), a malignant disorder of plasma cells, accounts for approximately 1% of all reported neoplasms and is the second most common haematologic cancer, accounting for 10%–15% of these malignancies [1], [2]. Patients with MM present with non-specific symptoms, such as fatigue, anemia, and bone pain, thus many patients have advanced disease at the time of diagnosis [3].

MM is a disease of the elderly, with an increase in the median age at diagnosis from 70 to 74 years over the last 50 years; only 15% of patients are <40 years of age at the time of diagnosis [4], [5]. The incidence of MM has increased over time. The reasons for the increase in the incidence of MM include a complete cancer registry report system, improved survival with new medical treatments, and the increasing elderly population with an longer life expectancy [4], [5]. A new challenge of this extended survival is secondary malignancies. The estimated incidence of secondary malignancies, especially hematologic malignancies, ranges between 1% and 15% [6]–[12]. Compared with Western countries, the incidence of MM is relatively low in Asia [13]–[15]; however, the incidence of MM has been increasing in Taiwan [16]. In addition, previous studies which have focused on the risk of secondary cancer have mostly evaluated Western populations [9], [17], [18]; indeed, there was limited data involving Asian populations. Therefore, the objectives of the current study were to determine the time trends in the incidence of MM between 1997 and 2009, and to investigate the association between MM and secondary tumours in Taiwan.

## Materials and Methods

### Dataset Sources

The National Health Research Institutes (NHRI) cooperates with the Bureau of National Health Insurance (BNHI) to establish several data files annually using the NHI claims data for public use. This insurance program was integrated in 1996 and had a coverage rate of approximately 99% of the 23.7 million people in Taiwan in 2009 [19]. The present study used NHIR subset data files of catastrophic illnesses, as well as the entire insured registration file. We used encrypted unique personal identifications linked files to obtain the chronologic medical history of each individual. Diseases were coded based on the International Classification of Disease Diagnoses, Ninth Revision of Clinical Modification (ICD-9-CM). The scrambled personal identifications secured confidentiality, preventing ethical violation from using the claims data. According to personal information protection, the identification was scrambled by BNHI before release to each researcher, and the study was exempted from ethical review. The study was approved by the Institutional Review Board (IRB) of China Medical University. The local IRB approval number was CMU-REC-101-012.

### Study Subjects

For this retrospective cohort study, we identified 3970 patients newly diagnosed with MM (ICD-9-CM code 203) between 1997 and 2009 from the registry of catastrophic illness, a sub-data set of the NHI research database. The diagnosis date was defined as the index date used to initiate follow-up for person-years measurement. Patients with MM and any other type of cancer recorded before the index date were excluded. The comparison subjects were randomly selected from all NHI beneficiaries and frequency-matched with the MM patients at a 4∶1 ratio for age, gender, and year that MM was diagnosed. Similarly, individuals with a history of MM and/or cancer at the baseline were excluded from the comparison cohort. We thus included 15880 non-MM subjects in the comparison cohort.

### Outcome Measures

All study subjects were followed until they were diagnosed with a secondary malignancy, which were identified from the registry of catastrophic illnesses. The duration of follow-up was estimated from the index date to the date of the secondary cancer being diagnosed, death, or 31 December 2009, whichever came first. We further estimated the incidence and risks for both cohorts of two types of secondary malignancies, as follows: hematologic malignancies (ICD-9-CM codes 201, 202, 204, 205, and 206); and solid tumors (ICD-9-CM codes 147, 150, 151, 153, 154, 155, 156, 157, 162, 173, 174, 185, 189, and 193).

### Statistical Analysis

**Figure 1:**
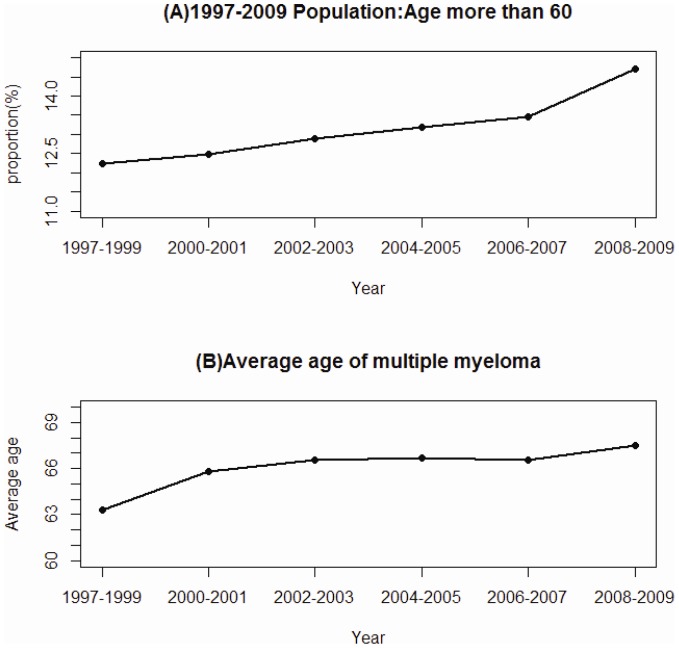
Proportion of insured population aged >60 years measured chronologically (A), and mean ages of patients with newly diagnosed multiple myeloma (B) for the period of 1997–2009.

**Figure 2:**
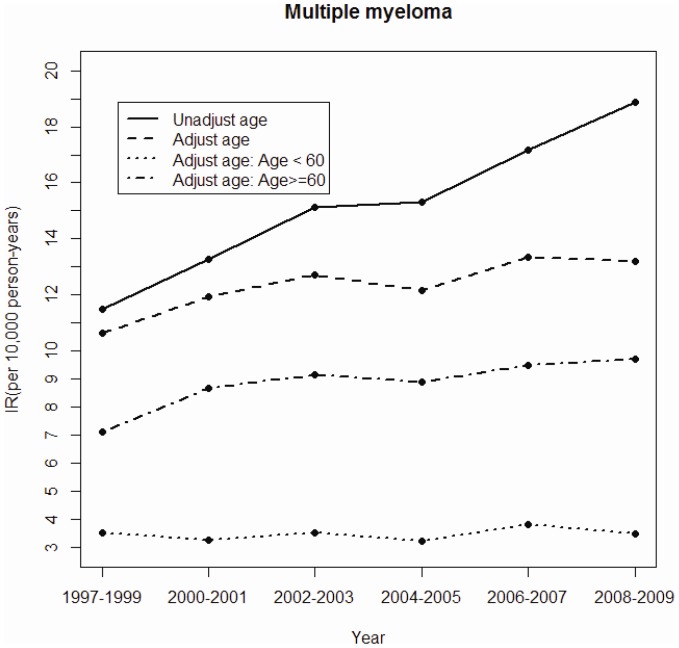
Unadjusted and adjusted overall incidence rates and age-specific adjusted incidence of multiple myeloma for the period of 1997–2009.

**Figure 3:**
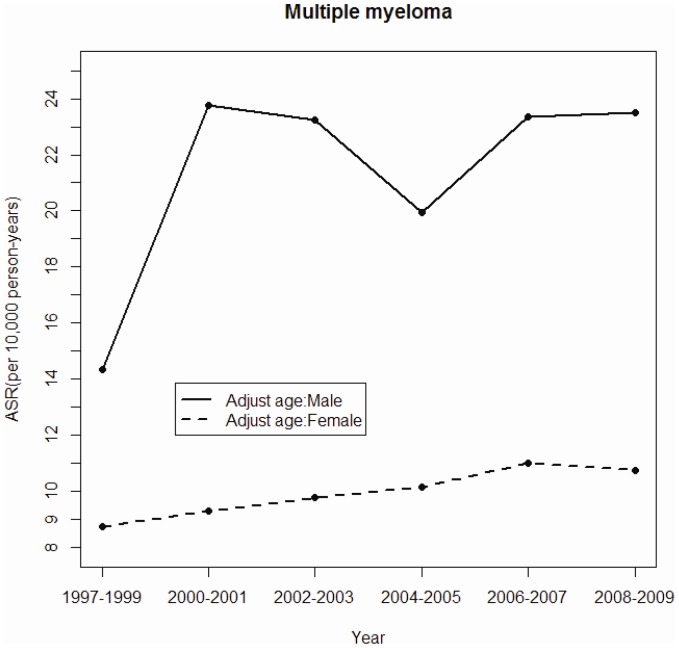
Gender-specific age-adjusted incidence of multiple myeloma for the period of 1997–2009.

We performed two sets of data analyses. One set was to demonstrate the annual changes of aging and the annual overall incidence of MM for the whole insured population. Data analysis was, therefore, first to depict the trend of aging for the whole population by measuring annually the proportion of population who aged >60 years from 1997 to 2009 [Figure 1(A)]. The mean age of patients with MM at the diagnosis was also measured annually between 1997 and 2009 and examined with simple correlation regression [Figure 1(B)]. Further the mean age of patients with MM at the diagnosis was also measured annually between 1997 and 2009. We plotted the annual incidence of MM for the whole insured population as the Kaplan-Meier estimates and examined with log-rank test after stratifying by age (<60 vs. ≥60 years) (Figure 2). The age standardization was performed using the 2000–2025 world population as the reference population. Gender-specific and age-specific (<60 years and >60 years) incidence were measured as well (Figure 3). The significance levels for the proportional trends in all figures were examined using Cochran-Armitage Trend test [20].

The other set of data analyses focused on measuring the incidence and risk of the secondary malignancy for the MM cohort and the comparisons. We measured the overall incidence, incidence by sex, age and tumor type (Hematologic tumors and Solid tumors) for both cohorts. The MM patients to comparisons incidence rate ratio (IRR) of secondary malignancies and 95% confidence interval (CI) was measured using Poisson regression analysis. In addition, we estimated the hazard ratio (HR) of the secondary malignancy and 95% CI using Cox proportional hazards regression analysis. We further tested the interaction between gender and MM and between age and MM by including a cross-product term in the model. SAS software (version 9.1; SAS Institute, Cary, NC, USA) was used for data analyses, with two-sided probability values <0.05 considered statistically significant.

## Results

Figure 1 (A) shows that the proportion of the insured population aged >60 years increased annually from 12.2% in 1997–1999 to 14.7% in 2008–2009 (p for time trend <0.0001). The mean age of the patients newly diagnosed with MM increased as well from 63.3 years in 1997–1999 to 67.5 years in 2008–2009 [Figure 1(B); p = 0.029].

The overall mean annual incidence of MM increased from 11.5 per 10,000 person-years in the period of 1997–1999 to 18.9 per 10,000 person-years in the period of 2008–2009 (p for time trend <0.0001) (Figure 2). The corresponding incidence rates after adjusting against the WHO population were 10.7 and 13.2 per 10,000 person-years (p for time trend <0.0001). The incidence was much greater in the older group, aged ≥60 years. There was no significant time variation in the younger group (p for time trend = 0.334). Figure 3 shows that the annual incidence of MM was much higher in men than in women (p for time trend, both <0.0001).

Table 1 shows the distribution of demographic status of our sample, both cohorts were similar in gender and age. The over all incidence of secondary malignancies in MM patients was slightly lower than the incidence of new malignancies in the comparison cohort, and not statistically significant (93.56 vs. 104.48 per 10,000 person-years, IRR = 0.90, 95% CI = 0.78–1.04; Table 2). However, the MM patients had a much higher incidence of hematologic malignancies than the incidence of new malignancies in the comparison cohort (47.20 vs. 4.09 per 10,000 person-years), with an IRR of 11.3 (95% CI = 10.3–12.4) and an adjusted HR of 13.0. The incidence rate of Hodgkin's disease, non-Hodgkin's lymphoma, myeloid leukemia, and lymphoblastic leukemia were all higher in the MM cohort. The risk of myeloid leukemia was greatest for MM patients with an adjusted HR of 23.9 (95% CI = 10.5–54.5). In contrast, the incidence of solid tumors was lower in MM patients than the non-MM cohort (47.1 vs. 98.7 per 10,000 person-years, IRR = 0.47, 95% CI = 0.39–0.57). The site-specific analysis of solid tumors showed a lower incidence for most sites in the MM cohorts than the comparison group.

**Table 1:** Demographic characteristics of cohorts with and without multiple myeloma.

	Multiple Myeloma		
	Yes (n = 3970)	No (n = 15880)	p-value
Age, mean±SD	66.11±13.0	66.10±12.95	0.98#
Age group (years)			
< = 29	45(1.13)	180(1.13)	1
30–44	164(4.13)	656(4.13)	
45–59	889(22.4)	3556(22.4)	
60–74	1793(45.2)	7172(45.2)	
> = 75	1079(27.2)	4316(27.2)	
Gender			
Female	1599(40.3)	6396(40.3)	1
Male	2371 (59.7)	9484(59.7)	

# p value was evaluated by Student’s *t*-test.

**Table 2:** Incidence, rate ratio, and adjusted hazard ratio of secondary cancer evaluated between patients with and without multiple myeloma.

	Multiple myeloma			Non-multiple myeloma				
	Event	PY	Rate#	Event	PY	Rate#	IRR*(95% CI)	Adjusted HR† (95% CI)
Total secondary cancer	71	7589	93.6	829	79345	104.5	0.90(0.78, 1.04)	1.08(0.84, 1.38)
*Hematologic tumor*	35	7416	47.2	33	80703	4.09	11.3(10.3, 12.4)	13.0(7.79, 21.6)**
Hodgkin's lymphoma	2	7700	2.60	0	80728	0.00	−	−
Non-Hodgkin's lymphoma	15	7627	19.67	21	80711	2.60	7.56(6.81, 8.39)**	7.72(3.83, 15.6)**
Myeloid leukemia	17	7668	22.17	11	80722	1.36	16.3(14.7, 18.0)**	23.9(10.5, 54.5)**
Lymphoblastic leukemia	1	7701	1.30	1	80727	0.12	10.5(9.31, 11.8)**	12.9(0.77, 215.7)
Monocytic	0	7703	0.00	0	80728	0.00	−	−
*Solid tumor*	36	7651	47.05	796	79369	98.66	0.47(0.39, 0.57)**	0.57(0.40, 0.79)**
Thyroid	1	7699	1.30	12	80679	1.49	0.87(0.70, 1.08)	0.71(0.09, 5.60)
Hepatoma	7	7700	9.09	133	80595	16.49	0.55(0.45, 0.68)**	0.66(0.30, 1.41)
Skin	2	7698	2.60	30	80654	3.72	0.70(0.56, 0.87)**	0.94(0.22, 4.11)
Lung	3	7702	3.90	144	80613	17.88	0.22(0.16, 0.30)**	0.28(0.09, 0.87)*
Colorectal	4	7700	5.19	102	80557	12.67	0.41(0.32, 0.53)**	0.52(0.19, 1.43)
Prostate	4	7692	5.20	85	80487	10.55	0.49(0.39, 0.62)**	0.61(0.22, 1.69)
Stomach	5	7693	6.50	78	80538	9.68	0.67(0.55, 0.82)**	0.81(0.32, 1.02)
Gastric	2	7702	2.60	81	80589	10.05	0.26(0.19, 0.35)**	0.37(0.09, 1.51)
Breast	3	7695	3.90	47	80590	5.82	0.67(0.54, 0.83)**	0.65(0.20, 2.12)
Kidney	1	7702	1.30	24	80692	2.97	0.44(0.33, 0.57)**	0.52(0.07, 3.88)
Pancreatic	1	7702	1.30	21	80716	2.60	0.50(0.38, 0.65)**	0.57(0.07, 4.33)
Esophagus	1	7702	1.30	21	80706	2.60	0.50(0.38, 0.65)**	0.49(0.06, 3.68)
Gallbladder	1	7702	1.30	10	80709	1.24	1.05(0.86, 1.28)	1.04(0.13, 8.40)
Nasopharyngeal	1	7697	1.30	8	80714	0.99	1.31(1.09, 1.57)**	1.08(0.13, 8.89)

# Rate, incidence rate, per 10,000 person-years.

* IRR, multiple myeloma cohort to non-multiple myeloma cohort incidence rate ratio, per 10,000 person-years.

† Adjusted HR, Cox model measured adjusted hazard ratio by adjustment for age and gender.

* p<0.05,

** p<0.01.

ICD-9-CM: hematologic malignancy, 201, 202, 204, 205, 206; Hodgkin's disease, 201; non-Hodgkin's lymphoma, 202; lymphoblastic leukemia, 204.0; myeloid leukemia, 205.0; monocytic leukemia, 206; solid tumor, 147, 150, 151, 153, 154, 155, 156, 157, 162, 173, 174, 185, 189, 193; nasopharyngeal cancer, 147; esophagus cancer, 150; gastric cancer, 151; colorectal cancer, 153; stomach cancer, 154; hepatoma, 155; gallbladder cancer, 156; pancreatic cancer, 157; lung cancer, 162; skin cancer, 173; breast cancer, 174; prostate cancer, 185; kidney cancer, 189; and thyroid cancer, 193.

We further evaluated the gender-specific and age-specific risk for hematologic malignancies alone. Results showed that the IRR of hematologic malignancies was higher in men than in women among patients with MM, but not in the non-MM cohort (Table 3). However, the gender specific HR for MM compared to non-MM cohort was approximately 13.0 with no significant difference between genders after adjusting for age. The age-specific analysis shows that the younger MM patients (<60 years of age) were at much greater risk of having hematologic malignancies than older groups. Compared non-MM patients, the younger MM patients had an IRR of 39.4 (95% CI = 31.7, 49.0) and an adjusted HR of 47.9 (95% CI = 15.9–144.0). In the interaction analysis, age significantly modified the association between MM and hematologic malignancy (p-values for interaction 0.0018).

**Table 3:** Gender-and age-specific incidence, incidence rate ratio and adjusted hazard ratio of hematologic malignancy by gender and age between cohorts with and without multiple myeloma.

	Multiple myeloma			Non-multiple myeloma					
	Event	PY	Rate#	Event	PY	Rate#	IRR*(95% CI)	Adjusted HR† (95% CI)	p-value&
Gender									0.6641
Female	13	3160	41.14	14	33404	4.19	9.82(8.47, 11.4)**	13.3(5.92, 29.8)**	
Male	22	4429	49.67	19	47300	4.02	12.4(10.9, 14.0)**	12.7(6.61, 24.5)**	
Age (years)									0.0018
<60	19	2951	64.38	4	24487	1.63	39.4(31.7, 49.0)**	47.9(15.92, 144.0)**	
60–74	12	3285	36.53	17	39132	4.34	8.41(7.27, 9.73)**	11.7(5.21, 26.1)**	
> = 75	4	1352	29.59	12	17085	7.02	4.21(3.34, 5.31)**	3.59(1.08, 11.9)*	

# Rate, incidence rate, per 10,000 person-years.

* IRR, multiple myeloma cohort to non-multiple myeloma cohort incidence rate ratio, per 10,000 person-years.

† Adjusted HR, Cox model measured adjusted hazard ratio by adjustment for age and gender.

* p<0.05,

** p<0.01.

& p-value for interaction.

## Discussion

The advancing incidence of MM worldwide might be due to the aging populations, and improved diagnosis and treatments [4], [21]. In Taiwan the proportion of the population >60 years of age increased from 12.2% in 1997–1999 to 14.7% in 2008–2009, and the incidence of MM increased significantly in the elderly people. The present study revealed the age-adjusted incidence of MM increased in Taiwan from 10.7 per 10,000 in 1997 to 13.2 per 10,000 in 2009. The increasing trend of MM in Taiwan is less strong than that in Western countries [2], [22]. Of note, the population in Taiwan is aging more rapidly than the Western countries. Specifically, it took 115 years for France people become aged, 85 years for Swedish, and 73 years for the American. On the other hand, it took only 24 years for Taiwan to become an aged society. By 2025, Taiwan will become a super-aged society, with >20% of its population considered elderly [23]. Some epidemiologic studies have suggested that the increased incidence of MM might be explained by toxins, food sources, and environmental pollutants. Environmental pollution, such as polychlorinated biphenyls (PCBs), polychlorinated dibenzo-p-dioxins (PCDDs), and polychlorinated dibenzofurans (PCDFs) may play a role in the pathogenesis of MM [24]–[27]. There are higher levels of pollutants (PCBs, PCDDs, and PCDFs) in elderly subjects than younger subjects according to studies from Taiwan [28], [29].

The data of the present study showed a much higher risk of secondary hematologic malignancies, especially myeloid leukemia and non-Hodgkin's lymphoma, in MM patients. The findings were in agreement with previous results based on Western populations [6]–[12], [30], [31]. In the 1997–2009 study period, there were 1.8% (n = 71) and 0.9% (n = 35) of 3970 patients with MM had secondary malignancies and secondary hematologic malignancies, respectively. Compared with the non-MM comparison cohort, MM patients had a 13- and 24-fold increased risk of developing secondary hematologic malignancies and myeloid leukemia, respectively. The duration between the diagnosis of MM and the occurrence of a secondary malignancy, and between the diagnosis of MM and the occurrence of a secondary hematologic malignancy were similar (1.9 years). The higher incidence risk and shorter duration of time to diagnosis of a secondary hematologic malignancy in the present research differed from the results of a Western population-based study [12]. The higher risk of secondary malignancies in younger cancer patients than other populations has been documented with respect to other cancers, including ovarian borderline tumors, breast cancer, and non-Hodgkin's lymphoma [32]–[35]. The risk of a second malignancy in patients with MM might result from genetic susceptibility, various medical treatments, or environmental co-risk factors of secondary malignancies. Amongst the recorded hematologic malignancies, such as non-Hodgkin's lymphoma (NHL), there was a higher risk of leukemia, Hodgkin's lymphoma, colorectal cancer, and lung cancer in patients <45 years of age [35]. The current study showed an age-related risk of secondary hematologic cancer; the risk decreased with age for patients with MM, which has not been documented in another population-based study.

The mean survival time of MM patients who died within this study period was 0.33 years (SD = 0.02), and the mean follow-up (survival) time of MM patients was 2.18 years (SD = 0.04). According to the current study, secondary hematologic malignancy occurred approximately 2 years after MM was diagnosed. With novel MM therapies, an increasing number of patients are living longer [4]. In the present study, the mean survival time of patients with MM increased from 1.39 years in the 1997–1999 periods to 1.58 years in the 2003–2005 periods. Consequently, clinical hematologists will face an increased incidence of secondary hematologic malignancy in patients with MM in the future.

The biologic mechanisms underlying acute myelocytic leukemia (AML) and myelodysplastic syndrome (MDS) following MM is still debatable, but might be explained by treatment-related factors, including lenalidomide use, cumulative melphalan dose, duration of melphalan therapy, a combination of factors [7]–[11], [31], or disease-related factors in secondary cancers [12], [36]. Furthermore, polymorphisms in germline genes may contribute to subsequent cancers [37], [38].

There were some limitations in the current study. We could not acquire detailed information of clinical or pharmacy treatment data. Therefore, we did not evaluate the importance of contributing factors to secondary malignancies, such as dose and duration of lenalidomide or melphalan or disease stage, nor did we differentiate the molecular subtype of MM form the database. Nevertheless, the advantage of the present study was the 12-year observation period and nationwide population-based data with access to standardized health care during the entire study period. The study design ensured case ascertainment and uniform up-to-date diagnostic criteria of all study subjects. Further, we eliminated recall bias and achieved generalized findings.

This nationwide population-based research shows that patients with MM are at a lower risk of having over all secondary malignancies than general population of having primary cancer because of lower incidence of solid tumor. The MM patients are at a much higher risk for hematologic malignancies, with younger patients are particularly vulnerable to the impact. The present study also provides new information for the Asian population that will help guide clinical and health service planning in the treatment of MM patients.
